# Normal Values of QT Variability in 10-s Electrocardiograms for all Ages

**DOI:** 10.3389/fphys.2019.01272

**Published:** 2019-10-04

**Authors:** Marten E. van den Berg, Jan A. Kors, Gerard van Herpen, Michiel L. Bots, Hans Hillege, Cees A. Swenne, Bruno H. Stricker, Peter R. Rijnbeek

**Affiliations:** ^1^Department of Medical Informatics, Erasmus MC – University Medical Center Rotterdam, Rotterdam, Netherlands; ^2^Julius Center for Health Sciences and Primary Care, University Medical Center Utrecht, Utrecht University, Utrecht, Netherlands; ^3^Department of Cardiology, University Medical Center Groningen, Groningen, Netherlands; ^4^Department of Cardiology, Leiden University Medical Center, Leiden, Netherlands; ^5^Department of Epidemiology, Erasmus MC – University Medical Center Rotterdam, Rotterdam, Netherlands; ^6^Department of Internal Medicine, Erasmus MC – University Medical Center Rotterdam, Rotterdam, Netherlands; ^7^Inspectorate of Health Care, Utrecht, Netherlands

**Keywords:** electrocardiography, normal limits, QT variability, heart-rate variability, children, elderly

## Abstract

**Aims:** QT variability is a promising electrocardiographic marker. It has been studied as a screening tool for coronary artery disease and left ventricular hypertrophy, and increased QT variability is a known risk factor for sudden cardiac death. Considering that comprehensive normal values for QT variability were lacking, we set out to establish these in standard 10-s electrocardiograms (ECGs) covering both sexes and all ages.

**Methods:** Ten-second, 12-lead ECGs were provided by five Dutch population studies (Pediatric Normal ECG Study, Leiden University Einthoven Science Project, Prevention of Renal and Vascular End-stage Disease Study, Utrecht Health Project, Rotterdam Study). ECGs were recorded digitally and processed by well-validated analysis software. We selected cardiologically healthy participants, 46% being women. Ages ranged from 11 days to 91 years. After quality control, 13,828 ECGs were available. We assessed three markers: standard deviation of QT intervals (SDqt), short-term QT variability (STVqt), and QT variability index (QTVI).

**Results:** For SDqt and STVqt, the median and the lower limit of normal remained stable with age. The upper limit of normal declined until around age 45, and increased strongly in the elderly, notably so in women. This implies that a subset of the population, small enough not to have appreciable effect on the median, shows a high degree of QT variability with a possible risk of arrhythmias or worse, especially in women. Otherwise, sex differences were negligible in all three measurements. For QTVI, median, and normal limits decreased until age 20, and steadily went up afterwards except for the lower limit of normal, which flattens off after age 65.

**Conclusion:** We report the first set of normal values for QT variability based on 10-s ECGs, for all ages and both sexes.

## Introduction

QT variability is a measure of the spontaneous fluctuations in the duration of the QT interval on the electrocardiogram (ECG). These fluctuations are thought to be the result of local variations in cardiac repolarization (Berger et al., 1997), and to reflect cardiac electrical instability (Baumert et al., 2016). For this reason, QT variability has been studied in relation to ventricular arrhythmias (Haigney et al., 2004) and sudden cardiac death (SCD) (Piccirillo et al., 2007). Moreover, increased QT variability was found to be a useful screening tool for coronary artery disease and left ventricular hypertrophy (Schlegel et al., 2010), which are important underlying causes of SCD (Albert et al., 1996; Haukilahti et al., 2019). The effects of cardiac drugs on QT variability have also been studied: β-blockers decrease QT variability (except sotalol), while digoxin and β-sympathicomimetic drugs increase QT variability (Niemeijer et al., 2014). The most commonly used QT-variability markers are the standard deviation of QT intervals (SDqt), short-term QT variability (STVqt), and the QT variability index (QTVI) (Niemeijer et al., 2014; Baumert et al., 2016). Definitions of these markers are given in the paragraph on QT variability measurement below.

QT variability has been measured on ECG recordings down to 10 s, and up to 24 h (Niemeijer et al., 2014; Baumert et al., 2016). Because the standard 10-s ECG is cheap, non-burdening, and in ubiquitous use, it would pay off to have normal values for QT variability based on 10-s ECGs. A position paper by Baumert et al. presented a meta-analysis of previous QT-variability studies including healthy participants (Baumert et al., 2016). This meta-analysis only showed study-specific average values for SDqt and QTVI, but did not stratify on sex or age (other than distinguishing between adults, children, and infants). Some of the studies that were reviewed reported age-specific normal limits (Yeragani et al., 2000; Piccirillo et al., 2006; Boettger et al., 2010; Kusuki et al., 2011; Baumert et al., 2013), but all these studies were done on small sample sizes and none used 10-s ECGs.

In the present study we provide normal values for the commonly used QT-variability markers SDqt, STVqt, and QTVI, based on a large set of standard 12-lead, 10-s ECGs of cardiologically healthy persons, covering both sexes and all ages.

## Methods

### Study Population

We combined data from five population studies conducted in the Netherlands. The 10-s, 12-lead ECGs from these studies were digitally recorded and stored at sampling rates of at least 500 Hz, up to 1,200 Hz in the pediatric group. All data were anonymized. The following studies were included:

1. Pediatric Normal ECG Study (Rijnbeek et al., 2001): The population of this study consists of 1,912 children, their ages ranging from 11 days to 16 years. The children were recruited in the year 2000 at three children's health centers, three primary schools, and one secondary school in the city of Rotterdam. The childrens' height and weight, measured before ECG recording, corresponded well with the Dutch growth standard (Rijnbeek et al., 2001). ECGs were recorded with a portable PC-based acquisition system (Cardio Control, Delft, The Netherlands).
2. Leiden University Einthoven Science Project (Scherptong et al., 2008): The population of this study contains 787 medical students of Leiden University. The ages of the participants ranged between 17 and 29 years, and all attested to be in good health. The ECGs were recorded from 2005 until 2007 with Megacart electrocardiographs (Siemens, Erlangen, Germany).
3. Prevention of Renal and Vascular End-stage Disease (PREVEND) Study (de Jong et al., 2003): This study, which started in 1997, aims to investigate the natural course of microalbuminuria and its relation to renal and cardiovascular disease in the general population. The PREVEND population consists of 8,592 participants aged 28–75 years, from the city of Groningen. Medical records, including medication use, were available for all participants. ECGs were recorded with CardioPerfect equipment (Welch Allyn, USA).
4. Utrecht Health Project (Grobbee et al., 2005): This ongoing study started in 2000 in Leidsche Rijn, a newly developed residential area of Utrecht. All new inhabitants were invited by their general practitioner to participate. The population of this study consists of 6,542 participants with ages ranging from 17 to 85 years. Written informed consent was obtained and an individual health profile was made by dedicated research nurses. Baseline assessment included physical examination, ECG, blood tests, and interview-assisted questionnaires. Pharmacy records were used to obtain information about medication. ECGs were recorded with CardioPerfect equipment (Welch Allyn, USA).
5. Rotterdam Study (Hofman et al., 1991): This study, which started in 1990, investigates determinants of a number of age-related disorders in an elderly population, prominently among them cardiovascular disease. The first two cohorts of the Rotterdam Study population consist of 10,994 inhabitants of Ommoord, a suburb of Rotterdam, aged 55 years or older. Participants were visited at home for an interview and were subsequently examined at the research center. Detailed information was collected on health status, medical history, and medication use. ECGs were recorded with an ACTA electrocardiograph (Esaote, Florence, Italy).

From these five populations, totaling 28,827 participants, we excluded individuals with proof or suspicion of cardiac disease. Reasons for exclusion were, therefore, a history of myocardial infarction, heart failure, coronary bypass surgery, coronary angioplasty, or pacemaker implantation. Further exclusion criteria were hypertension and diabetes mellitus. Hypertension was defined as a systolic blood pressure ≥160 mmHg or a diastolic blood pressure ≥100 mmHg or use of antihypertensive medication, including beta-blockers. Diabetes mellitus was defined as a non-fasting serum glucose ≥11 mmol/l or use of glucose-lowering drugs. After applying these criteria, 15,248 individuals remained available.

This study was approved by the Medical Ethics Committee of the Erasmus University Medical Center. Since all data were anonymized and retrospectively collected, individual informed consent from the participants in our study was not required according to Dutch legislation.

### ECG Quality Control

All ECGs were manually checked. We removed ECGs with disturbances that could potentially affect accurate measurement of the QT interval, such as excessive noise, excessive baseline wander, and sudden baseline jumps or spikes. We also removed ECGs with premature ventricular beats, premature supraventricular beats, and second or third degree atrioventricular block. This resulted in a final 13,828 ECGs for analysis.

### QT-Variability Measurement

The ECGs were processed by our Modular ECG Analysis System (MEANS), an ECG computer program that has been evaluated extensively (van Bemmel et al., 1990; Willems et al., 1991). MEANS signals excessive noise and baseline wander, recognizes ectopic beats and the various forms of AV block. It computes one representative averaged beat over all beats of each of the 12 leads and identifies within these averaged beats the P, QRS, and T waves with their points of onset and offset for the 12 leads simultaneously. For the special case of QT-interval measurement fiducial segment averaging (FSA) was applied, a technique that exploits the correlation between signal segments from individual beats (Ritsema van Eck, 2002). An FSA implementation in combination with MEANS has been described and evaluated extensively before (Rijnbeek et al., 2017). Briefly, MEANS first determines the locations of the individual QRS complexes and provisional fiducial points (i.e., the onsets of the QRS complexes and the ends of the T waves). Baseline wander is corrected using restricted splines, and a detection function consisting of the root-mean-square ECG signal is computed. Second, the fiducial point in each individual beat is shifted in an iterative procedure until maximum correlation is achieved between a small signal segment of the detection function around this fiducial point and the average of the segments around the fiducial points of the remaining complexes. The amount of shifting is retained and constitutes the individual beat variation. Finally, the QT interval for each beat is calculated from the shifts of the initial QRS onset and T end.

We calculated the QT-variability markers SDqt, STVqt, and QTVI (Niemeijer et al., 2014). SDqt is defined as the standard deviation of the QT intervals. STVqt is defined as the average of the absolute differences between the QT intervals of subsequent beats:

(1) $$\begin{array}{r} \text{STVqt}=\sum_{i=1}^{n}\frac{\left|QT_{i+1}-QT_{i}\right|}{n\sqrt{2}} \end{array}$$

where *n* is the number of differences.

QTVI normalizes the variance of the QT intervals (*QT*_*v*_) for mean QT (*QT*_*m*_) squared, and the variance of the heart rate (*HR*_*v*_) for mean heart rate (*HR*_*m*_) squared, and is defined as:

(2) $$\begin{array}{r} \text{QTVI}=log_{10}\left[\left(\frac{QT_{v}}{QT_{m}^{2}}\right)/\left(\frac{HR_{v}}{HR_{m}^{2}}\right)\;\right] \end{array}$$

In 11 ECGs all QT intervals were measured as identical, producing SDqt and STVqt values of zero. However, if the variance of QT is zero, QTVI cannot be computed because the logarithm of zero is undefined. These cases are addressed by setting the QTVI to −3.60, the lowest QTVI value that occurred in our data.

### Estimation of Normal Values

We used the Box-Cox *t* distribution in a semi-parametric model for location, scale, and shape to estimate centile curves (Cole and Green, 1992). The Box-Cox *t* distribution allows for modeling of the distribution of the median, skewness, and kurtosis as functions of age. The lower limit of normal (LLN) was defined as the 2nd percentile and the upper limit of normal (ULN) as the 98th percentile. The Box-Cox *t* distribution was implemented with the *lms* function of the R-package *gamlss*, and the normal values for all age categories were estimated using the *predict.gamlss* function of the *gamlss* package. The normal values of a given age category were estimated for the central age in that category. For example, normal values for the category of 30–39 years are based on the estimated values for participants aged 35 years.

Differences in LLN, median, and ULN between men and women were tested per age group, using non-parametric estimates and a bootstrap approach with 5,000 bootstrap samples (Johnson and Romer, 2016).

## Results

**Figure 1:**
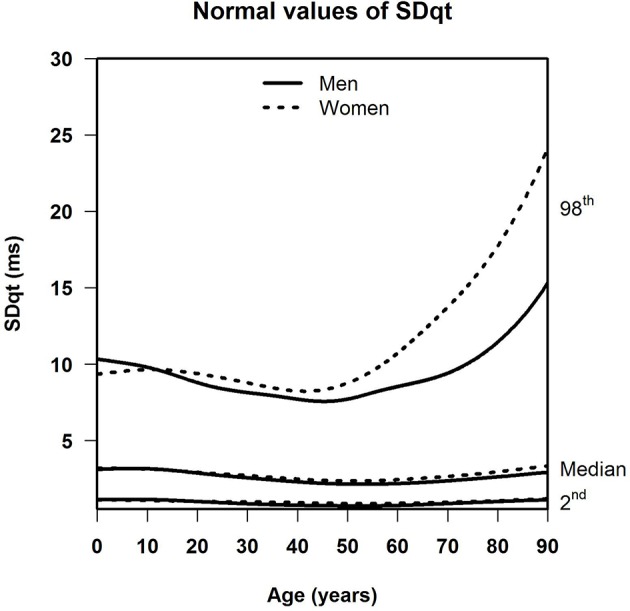
Median, 2nd, and 98th percentiles of SDqt for all ages in men and women.

**Figure 2:**
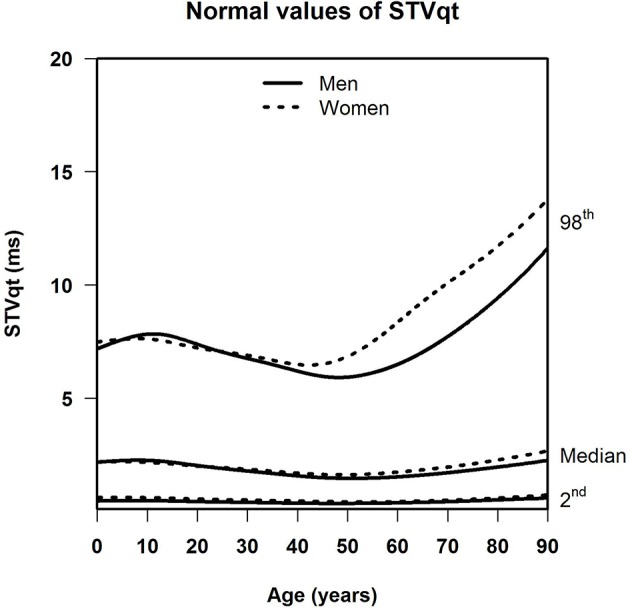
Median, 2nd, and 98th percentiles of STVqt for all ages in men and women.

**Figure 3:**
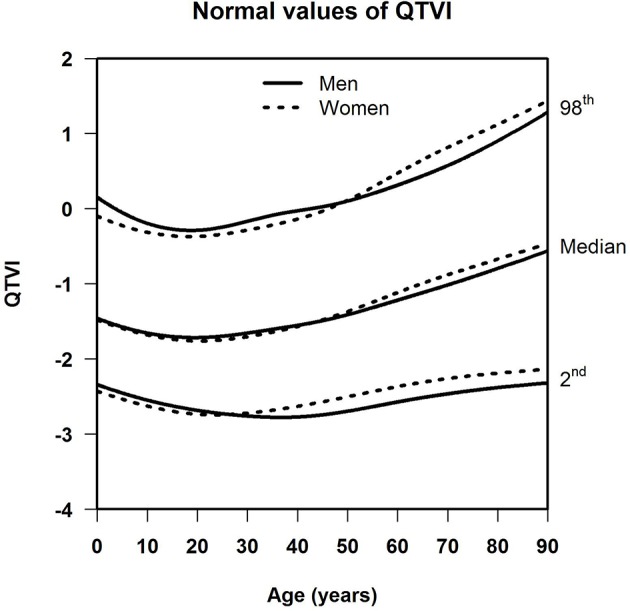
Median, 2nd, and 98th percentiles of QTVI for all ages in men and women.

Table 1 shows the number of available ECGs grouped by sex and age category. Most age groups had more than 100 ECGs, only the age groups below 6 months or above 90 years contained fewer ECGs. Table 2 gives the age-dependent median, ULN, and LLN of SDqt, stratified by sex. Table 3 shows these statistics for STVqt and Table 4 for QTVI. Additional percentile values for the three markers are given in Supplementary Tables 1–3. Figures 1–3 show continuous age-dependent curves of the median and the normal limits of SDqt, STVqt, and QTVI, respectively.

**Table 1:** The study population stratified on sex and age groups.

**Age group**	**No. of boys/men**	**No. of girls/women**	**Total**
Younger than 1 month	9	8	17
1–2 months	26	22	48
3–5 months	33	37	70
6–11 months	69	53	122
1–2 years	50	51	101
3–4 years	60	61	121
5–7 years	120	104	224
8–11 years	115	163	278
12–15 years	138	100	238
16–19 years	155	381	536
20–29 years	450	908	1,358
30–39 years	1,374	1,962	3,336
40–49 years	960	1,109	2,069
50–59 years	972	1,227	2,199
60–69 years	991	1,212	2,203
70–79 years	288	460	748
80–89 years	49	106	155
90 years and older	1	4	5
Total	5,860	7,968	13,828

**Table 2:** Normal values for SDqt (in ms) per age group and sex.

	**Median (2nd percentile; 98th percentile)**	
**Age category**	**Boys/men**	**Girls/women**
<1 month	3.11 (1.13; 10.32)	3.18 (1.11; 9.34)
1–2 months	3.11 (1.13; 10.32)	3.18 (1.11; 9.34)
3–5 months	3.11 (1.14; 10.31)	3.18 (1.11; 9.35)
6–11 months	3.12 (1.14; 10.29)	3.18 (1.11; 9.37^‡^)
1–2 years	3.13 (1.14; 10.23)	3.18 (1.11; 9.42)
3–4 years	3.15 (1.15; 10.14)	3.18 (1.10; 9.49)
5–7 years	3.17 (1.15; 10.01)	3.17 (1.10; 9.58)
8–11 years	3.15 (1.14; 9.79)	3.13 (1.08; 9.64)
12–15 years	3.07 (1.10; 9.43)	3.06 (1.05; 9.61)
16–19 years	2.94 (1.04; 8.99)	2.96 (1.02; 9.47)
20–29 years	2.70 (0.93; 8.38)	2.81^†^(0.99; 9.10)
30–39 years	2.42 (0.80; 7.94)	2.59^†^ (0.96; 8.44^*^)
40–49 years	2.20 (0.74; 7.56)	2.39^‡^(0.90^*^; 8.31)
50–59 years	2.14 (0.72; 8.13)	2.37^‡^ (0.87; 9.57^*^)
60–69 years	2.26 (0.81; 8.90)	2.53^‡^ (0.91^*^; 12.16)
70–79 years	2.49 (0.94; 10.23)	2.79 (1.00; 15.52^*^)
80–89 years	2.77 (1.07; 13.09)	3.14^*^ (1.12; 20.57)

* P-value <0.05 for difference between men and women;

† P-value <0.01;

‡ *P-value <0.001*.

*SDqt, Standard deviation of QT interval*.

**Table 3:** Normal values for STVqt in milliseconds per age group and sex.

	**Median (2nd percentile, 98th percentile)**	
**Age category**	**Boys/men**	**Girls/women**
<1 month	2.18 (0.47; 7.18)	2.18 (0.63; 7.48)
1–2 months	2.18 (0.47; 7.19)	2.18 (0.63; 7.48)
3–5 months	2.18 (0.47; 7.21)	2.18 (0.63; 7.49)
6–11 months	2.19 (0.47; 7.25)	2.18 (0.63; 7.50^*^)
1–2 years	2.21 (0.47; 7.35)	2.19 (0.63; 7.53)
3–4 years	2.24 (0.48; 7.51)	2.19 (0.62; 7.58)
5–7 years	2.26 (0.48; 7.68)	2.19 (0.62; 7.62)
8–11 years	2.26 (0.48; 7.82)	2.17 (0.61; 7.61)
12–15 years	2.19 (0.47; 7.77)	2.11 (0.58; 7.49)
16–19 years	2.09 (0.45; 7.53)	2.04 (0.56; 7.31)
20–29 years	1.90 (0.42; 7.03)	1.93^‡^ (0.52; 7.05)
30–39 years	1.68 (0.38; 6.49)	1.78 (0.48^†^; 6.67)
40–49 years	1.49 (0.36; 5.96)	1.63 (0.44; 6.51)
50–59 years	1.48 (0.37; 6.12)	1.66^‡^ (0.42; 7.49^‡^)
60–69 years	1.61 (0.41; 7.03)	1.84^†^ (0.46; 9.26^†^)
70–79 years	1.83 (0.47; 8.52)	2.10 (0.54; 10.86)
80–89 years	2.10 (0.55; 10.45)	2.46 (0.66; 12.68)

* P-value <0.05 for difference between men and women;

† P-value <0.01;

‡ *P-value <0.001*.

*STVqt, Short-term variability of the QT interval*.

**Table 4:** Normal values for QTVI per age group and sex.

	**Median (2nd percentile; 98th percentile)**	
**Age category**	**Boys/men**	**Girls/women**
<1 month	−1.47 (−2.35; 0.14)	−1.49 (−2.44; −0.11)
1–2 months	−1.47 (−2.35; 0.14)	−1.49 (−2.44; −0.11)
3–5 months	−1.48 (−2.36; 0.12)	−1.50 (−2.45; −0.12)
6–11 months	−1.49 (−2.37; 0.11)	−1.51 (−2.46; −0.13^*^)
1–2 years	−1.52 (−2.40; 0.05)	−1.54 (−2.48; −0.16)
3–4 years	−1.56 (−2.44; −0.02)	−1.58 (−2.52; −0.21)
5–7 years	−1.61 (−2.49; −0.11)	−1.63 (−2.57; −0.26)
8–11 years	−1.66 (−2.56; −0.20)	−1.69 (−2.63; −0.32)
12–15 years	−1.70 (−2.62; −0.27)	−1.74 (−2.69; −0.36)
16–19 years	−1.72 (−2.67; −0.30)	−1.76 (−2.73; −0.38)
20–29 years	−1.70 (−2.74; −0.25)	−1.75 (−2.75; −0.35)
30–39 years	−1.61 (−2.78; −0.10)	−1.65^*^ (−2.69; −0.23^*^)
40–49 years	−1.50 (−2.75; 0.02)	−1.49 (−2.58; −0.04)
50–59 years	−1.33 (−2.64; 0.19)	−1.25^*^ (−2.44; 0.28)
60–69 years	−1.12 (−2.52; 0.43)	−1.00^†^ (−2.32; 0.64^†^)
70–79 years	−0.92 (−2.43; 0.72)	−0.78 (−2.23; 0.96^*^)
80–89 years	−0.69 (−2.36; 1.08)	−0.58 (−2.17; 1.27)

* P-value <0.05 for difference between men and women;

† *P-value <0.01*.

*QTVI, QT variability index*.

The median and LLN of SDqt and STVqt remain relatively constant over age. The ULN of both markers slightly decreases until the age of 45, after which it strongly increases. The normal values of QTVI show a decrease until about 20 years, after which they start to rise again. Overall, sex differences are limited for STVqt and SDqt, with the exception of the ULN after the age of 45 where the female curve follows a steeper course than its male counterpart. Although statistically significant differences between men and women can be found for the median values above age 20, all differences are <0.4 ms and do not appear practically relevant. For QTVI, sex differences are minimal.

## Discussion

This is the first paper to report comprehensive normal values of QT variability based on 10-s, standard 12-lead ECGs. We report the median, LLN, and ULN of SDqt, STVqt, and QTVI across all ages and stratified on sex.

A paper by Baumert et al. presents a meta-analysis of normal values from 45 studies involving 1,954 adults with QTVI measurements and from 23 studies involving 1,190 adults with SDqt measurements (Baumert et al., 2016). Without taking age or ECG-recording duration into account, this meta-analysis found for men a mean QTVI of −1.6 and a mean SDqt of 3.3 milliseconds. The underlying studies of this meta-analysis differ widely in methodology. None of these studies is based on the standard 10-s ECG, typically ECG recordings of 256 s or more were used (Baumert et al., 2016). In children, there is a study by Kusuki et al. that reports normal values of 173 children without organic heart disease aged 0–7 years, using only lead CM5 on ECG recordings containing 120 beats (Kusuki et al., 2011). Their paper concludes that QTVI decreases from birth to age 7, which is in accordance with our findings. Unfortunately this study does not give the results in percentiles but in means and standard deviations. A study by Yeragani et al. using 10-min recordings found no significant difference in QTVI between 15 children aged 6–14 years and 34 normal adults aged 20–55 years (Yeragani et al., 2000). Assuming that these 10-min recordings have the same age-dependent pattern as our 10-s recordings, this may be explained by the children being on the descending limb of the QTVI curve, while the adults are at the same height on the ascending limb. There are three studies that track normal values for QTVI derived from 10- to 30-min recordings over a longer age range, between 20 and 90 years (Piccirillo et al., 2006; Boettger et al., 2010; Baumert et al., 2013). These studies, with 40, 131, and 143 healthy participants, show that QTVI increases with age, which concurs with our findings (Piccirillo et al., 2006; Boettger et al., 2010; Baumert et al., 2013). Further, SDqt was analyzed by Kraus et al., showing that SDqt is higher in women and that SDqt increases with age (Krauss et al., 2009); both findings are consistent with the results of this study. Kraus et al. used 24 h Holter recordings, and calculated hourly mean values for SDqt.

As no other study reports median, LLN and ULN values stratified on sex, full-scale comparisons of our results with those of other studies are without being. To the extent that comparisons are possible, our findings are in general agreement with those of previous studies that used longer recordings. The age- and sex-dependent normal values of QT variability reported here may serve to establish cut-off values for various clinical and pharmacological applications. For example, a drug associated with risk of ventricular arrhythmias might be withheld if the QT variability is above a certain limit.

In a previous review on the literature of QT variability, we surmised that QT-variability markers carry information about the risk of ventricular arrhythmias, SCD, and total mortality: the higher the QT variability, the higher the risk, perhaps indicating repolarization instability (Niemeijer et al., 2014). In this light, the large increase of the ULN of SDqt and STVqt in older age compared to the relatively stable median and LLN might point to a subset of the population at risk for arrhythmias or worse, notably so in women, where this increase is more pronounced.

Our study has a number of strengths. With 13,828 ECGs it is by far the largest study reporting normal values for QT variability. We include both men and women, and have a wide age coverage, from 11 days to 90 years. All ECGs were analyzed consistently and automatically with the well-validated MEANS program and FSA technique (Ritsema van Eck, 2002; Rijnbeek et al., 2017). This approach eliminates intra-observer bias and uses all 12 ECG leads, which limits dependency of QT variability on lead-dependent T-wave amplitudes. Moreover, while concerns have been raised about the reproducibility of QT variability when measured beat-by-beat (Feeny et al., 2014), FSA exploits the correlation between individual beats and has been shown to produce highly reproducible results (Rijnbeek et al., 2017).

Our study also has limitations. First, we had a relatively low number of ECGs in the extremes of the age distribution. For this reason, we did not give normal limits for ages higher than 90 years, and the normal limits of participants younger than 6 months should be used with caution. Second, the use of 10-s recordings prohibits measurement of QT variability over longer time intervals, as required for the detection of, e.g., respiratory modulation and diurnal changes. However, respiratory modulation was studied by Emori and Ohe, who conclude that it does not affect QT variability in healthy participants (Emori and Ohe, 1999). As for diurnal changes, a study by Yeragani et al. has shown that QT variability differs between the waking and sleeping hours, but also that it is fairly constant within either period (Yeragani et al., 2005). Therefore, if all ECGs are recorded in awake persons this should not materially affect the results. Third, 10-s ECGs may sometimes contain only a few QT intervals, rendering the QT-variability estimates less reliable than in longer recordings. Finally, race is not a category that is officially recognized in The Netherlands and thus our records did not contain this information. However, the populations from which we have recruited our participants may deemed to be white in the large majority. Further studies are needed to determine the possible effect of race on the normal limits of QT variability.

In conclusion, normal limits have been established for SDqt, STVqt, and QTVI derived from 10-s ECGs, using a consistent and automatic methodology for all ages and both sexes. We found strong age effects for all markers. These normal values should help both researchers and clinicians to decide upon cut-off values of abnormal QT variability in 10-s ECGs, improving the practical applicability of QT variability.

## Data Availability Statement

The datasets generated for this study are available on request to the corresponding author.

## Ethics Statement

This study was approved by the Medical Ethics Committee of the Erasmus University Medical Center. Since all data were anonymized and retrospectively collected, individual informed consent from the study participants was not required according to Dutch legislation.

## Author Contributions

MEB, JK, and PR: conception and design of the work. MEB, GH, MLB, HH, CS, and BS: data collection and cleaning. JK and PR: data processing. MEB, JK, GH, and PR: statistical analysis and interpretation. MEB: drafting of the manuscript. All authors: critical review of the manuscript.

### Conflict of Interest

The authors declare that the research was conducted in the absence of any commercial or financial relationships that could be construed as a potential conflict of interest.
