# Supplementary material for: Normal Values of QT Variability in 10-s Electrocardiograms for all Ages
Source: Front Physiol. 2019 Oct 4;10:1272. doi: 10.3389/fphys.2019.01272 (PMC6788348; doi:10.3389/fphys.2019.01272)
Supplement: Supplementary file 2 [file Table_2.pdf]

Supplementary Table 2: Percentiles of STVqt (in ms) in various age categories of men and women.

Percentiles for Men

Age	2nd	5th	10th	25th	50th	75th	90th	95th	98th
< 1 month	0.47	0.68	0.91	1.41	2.18	3.25	4.61	5.67	7.18
1 to 3 months†	0.47	0.68	0.91	1.41	2.18	3.26	4.62	5.68	7.19
3 to 6 months	0.47	0.68	0.91	1.42	2.18	3.26	4.63	5.70	7.21
6 to 12 months	0.47	0.68	0.91	1.42	2.19	3.27	4.64	5.72	7.25
1 to 3 years	0.47	0.69	0.92	1.43	2.21	3.31	4.70	5.79	7.35
3 to 5 years	0.48	0.69	0.93	1.44	2.24	3.35	4.78	5.90	7.51
5 to 8 years	0.48	0.70	0.94	1.46	2.26	3.40	4.86	6.02	7.68
8 to 12 years	0.48	0.69	0.93	1.45	2.26	3.41	4.90	6.10	7.82
12 to 16 years	0.47	0.67	0.90	1.41	2.19	3.33	4.82	6.02	7.77
16 to 20 years	0.45	0.64	0.86	1.33	2.09	3.18	4.62	5.80	7.53
20 to 30 years	0.42	0.59	0.79	1.22	1.90	2.90	4.25	5.36	7.03
30 to 40 years	0.38	0.54	0.71	1.09	1.68	2.56	3.79	4.84	6.49
40 to 50 years	0.36	0.50	0.66	0.98	1.49	2.26	3.36	4.34	5.96
50 to 60 years	0.37	0.51	0.66	0.98	1.48	2.22	3.32	4.34	6.12
60 to 70 years	0.41	0.56	0.72	1.07	1.61	2.44	3.69	4.90	7.03
70 to 80 years	0.47	0.64	0.82	1.21	1.83	2.82	4.35	5.83	8.52
80 to 90 years	0.55	0.74	0.94	1.38	2.10	3.29	5.17	7.04	10.45

Percentiles for Women

Age	2nd	5th	10th	25th	50th	75th	90th	95th	98th
< 1 month	0.63	0.81	1.02	1.46	2.18	3.24	4.65	5.80	7.48
1 to 3 months	0.63	0.81	1.02	1.46	2.18	3.24	4.65	5.80	7.48
3 to 6 months	0.63	0.81	1.02	1.46	2.18	3.24	4.66	5.81	7.49
6 to 12 months	0.63	0.81	1.02	1.47	2.18	3.25	4.66	5.81	7.50
1 to 3 years	0.63	0.81	1.02	1.47	2.19	3.25	4.67	5.83	7.53
3 to 5 years	0.62	0.81	1.02	1.47	2.19	3.26	4.69	5.86	7.58
5 to 8 years	0.62	0.81	1.02	1.47	2.19	3.26	4.70	5.88	7.62
8 to 12 years	0.61	0.80	1.00	1.45	2.17	3.23	4.67	5.85	7.61
12 to 16 years	0.58	0.77	0.97	1.41	2.11	3.15	4.56	5.73	7.49
16 to 20 years	0.56	0.74	0.93	1.36	2.04	3.04	4.41	5.56	7.31
20 to 30 years	0.52	0.70	0.88	1.29	1.93	2.89	4.21	5.33	7.05
30 to 40 years	0.48	0.64	0.82	1.19	1.78	2.66	3.90	4.97	6.67
40 to 50 years	0.44	0.59	0.75	1.09	1.63	2.45	3.64	4.71	6.51
50 to 60 years	0.42	0.58	0.75	1.10	1.66	2.52	3.85	5.14	7.49
60 to 70 years	0.46	0.63	0.82	1.22	1.84	2.83	4.42	6.06	9.26
70 to 80 years	0.54	0.73	0.94	1.39	2.10	3.25	5.12	7.06	10.86
80 to 90 years	0.66	0.88	1.11	1.63	2.46	3.82	6.03	8.30	12.68

†The term “to” specifies the upper limit in the sense of “less than”.
