# Supplementary material for: Normal Values of QT Variability in 10-s Electrocardiograms for all Ages
Source: Front Physiol. 2019 Oct 4;10:1272. doi: 10.3389/fphys.2019.01272 (PMC6788348; doi:10.3389/fphys.2019.01272)
Supplement: Supplementary file 3 [file Table_3.pdf]

Supplementary Table 3: Percentiles of QTVI in various age categories of men and women.

	Percentiles for Men								
Age	2nd	5th	10th	25th	50th	75th	90th	95th	98th
< 1 month	-2.35	-2.21	-2.08	-1.82	-1.47	-1.05	-0.59	-0.27	0.14
1 to 3 months†	-2.35	-2.22	-2.08	-1.82	-1.47	-1.05	-0.59	-0.27	0.14
3 to 6 months	-2.36	-2.22	-2.09	-1.83	-1.48	-1.06	-0.60	-0.28	0.12
6 to 12 months	-2.37	-2.23	-2.09	-1.83	-1.49	-1.07	-0.61	-0.30	0.11
1 to 3 years	-2.40	-2.26	-2.12	-1.86	-1.52	-1.10	-0.65	-0.34	0.05
3 to 5 years	-2.44	-2.30	-2.16	-1.90	-1.56	-1.15	-0.71	-0.41	-0.02
5 to 8 years	-2.49	-2.35	-2.21	-1.95	-1.61	-1.20	-0.77	-0.48	-0.11
8 to 12 years	-2.56	-2.41	-2.27	-2.00	-1.66	-1.26	-0.84	-0.56	-0.20
12 to 16 years	-2.62	-2.47	-2.32	-2.05	-1.70	-1.30	-0.89	-0.61	-0.27
16 to 20 years	-2.67	-2.51	-2.36	-2.08	-1.72	-1.32	-0.91	-0.63	-0.30
20 to 30 years	-2.74	-2.55	-2.38	-2.08	-1.70	-1.29	-0.87	-0.59	-0.25
30 to 40 years	-2.78	-2.56	-2.36	-2.02	-1.61	-1.18	-0.74	-0.45	-0.10
40 to 50 years	-2.75	-2.51	-2.29	-1.92	-1.50	-1.05	-0.62	-0.33	0.02
50 to 60 years	-2.64	-2.38	-2.14	-1.76	-1.33	-0.87	-0.44	-0.15	0.19
60 to 70 years	-2.52	-2.24	-1.99	-1.58	-1.12	-0.65	-0.20	0.08	0.43
70 to 80 years	-2.43	-2.11	-1.84	-1.40	-0.92	-0.42	0.06	0.36	0.72
80 to 90 years	-2.36	-2.00	-1.70	-1.21	-0.69	-0.15	0.35	0.68	1.08

	Percentiles for Women								
Age	2nd	5th	10th	25th	50th	75th	90th	95th	98th
< 1 month	-2.44	-2.27	-2.11	-1.83	-1.49	-1.11	-0.71	-0.45	-0.11
1 to 3 months	-2.44	-2.27	-2.11	-1.83	-1.49	-1.11	-0.72	-0.45	-0.11
3 to 6 months	-2.45	-2.28	-2.12	-1.84	-1.50	-1.12	-0.72	-0.46	-0.12
6 to 12 months	-2.46	-2.28	-2.13	-1.85	-1.51	-1.13	-0.73	-0.47	-0.13
1 to 3 years	-2.48	-2.31	-2.15	-1.87	-1.54	-1.16	-0.76	-0.50	-0.16
3 to 5 years	-2.52	-2.35	-2.19	-1.92	-1.58	-1.20	-0.81	-0.55	-0.21
5 to 8 years	-2.57	-2.40	-2.24	-1.96	-1.63	-1.26	-0.87	-0.60	-0.26
8 to 12 years	-2.63	-2.46	-2.30	-2.02	-1.69	-1.32	-0.93	-0.66	-0.32
12 to 16 years	-2.69	-2.51	-2.35	-2.07	-1.74	-1.37	-0.98	-0.71	-0.36
16 to 20 years	-2.73	-2.55	-2.38	-2.10	-1.76	-1.39	-1.00	-0.73	-0.38
20 to 30 years	-2.75	-2.56	-2.39	-2.10	-1.75	-1.37	-0.98	-0.70	-0.35
30 to 40 years	-2.69	-2.49	-2.32	-2.01	-1.65	-1.25	-0.85	-0.57	-0.23
40 to 50 years	-2.58	-2.37	-2.18	-1.86	-1.49	-1.08	-0.66	-0.39	-0.04
50 to 60 years	-2.44	-2.21	-2.01	-1.66	-1.25	-0.81	-0.37	-0.08	0.28
60 to 70 years	-2.32	-2.06	-1.83	-1.45	-1.00	-0.52	-0.05	0.26	0.64
70 to 80 years	-2.23	-1.94	-1.69	-1.27	-0.78	-0.27	0.23	0.56	0.96
80 to 90 years	-2.17	-1.85	-1.57	-1.10	-0.58	-0.03	0.50	0.85	1.27

†The term "to" specifies the upper limit in the sense of "less than".
